# Correction: User authentication system based on human exhaled breath physics

**DOI:** 10.1371/journal.pone.0320609

**Published:** 2025-03-20

**Authors:** The PLOS One Staff

## Notice of republication

This article was republished on February 21, 2025, to correct typesetting errors, the equation on page 10 is incorrect as the number 2 should be in the subscript. On page 15, the second-last paragraph: `UCS.ML’ should be replaced with `UCA.ML’. The publisher apologizes for the errors. Please download this article again to view the correct version. The originally published, uncorrected article and the republished, corrected articles are provided here for reference.

## Supporting information

S1 File Originally published, uncorrected article. (PDF)

S2 File Republished, corrected article. (PDF)
