# Supplementary material for: User authentication system based on human exhaled breath physics
Source: PLoS One. 2024 Apr 22;19(4):e0301971. doi: 10.1371/journal.pone.0301971 (PMC11034670; doi:10.1371/journal.pone.0301971)
Supplement: S1 Text — The supporting information for this research article includes: Part 1: A statistical description which describes the Multifractal Detrended Fluctuation Analysis (MFDFA) of human exhaled breath velocity time series; Part 2: Model library building procedure and model selection for the machine learning based algorithm. (PDF) [file pone.0301971.s001.pdf]

S1 Text: Supplementary material
User authentication system based on human exhaled breath physics

Mukesh Karunanethy[1], Rahul Tripathi[1], Mahesh V Panchagnula*[1], and Raghunathan Rengaswamy[2]

[1]Department of Applied Mechanics and Biomedical Engineering, Indian Institute of Technology Madras, Chennai, Tamil Nadu, India
[2]Department of Chemical Engineering, Indian Institute of Technology Madras, Chennai, Tamil Nadu, India

Part 1: Statistical description of the time series

In general, a statistical description would involve the representation of time series distributions in terms of the central moments. Such representative measures tend to vary within a non-stationary time series. They can be characterized by studying how these moments depend on time intervals within the time series itself, by investigating the scaling properties of the signal. For instance, the Hurst exponent, H (Hurst (1951)) parametrizes the effect of the statistics of time intervals on the standard deviation of the time signal. In the context of multifractal analysis, the generalized Hurst exponent H(q) is used for parameterization, where q is the order of the fluctuation function (Kantelhardt et al. (2002)). H(q) is also known as the $q-$order Hurst exponent. In our study, we focus on the multifractal properties of the time series, since interestingly, human exhaled breath has been found to display multifractality, based on our analysis, which will be discussed in this section. Fully developed turbulence is known to exhibit multifractality, as described by Sreenivasan (1991).

The multifractal nature of exhaled breath signals were investigated using the well-known technique called multifractal detrended fluctuation analysis (MFDFA) developed by Kantelhardt et al. (2002). It helps us to identify multifractal scaling properties as well as to detect long-range correlations in a time series. A detailed explanation of the theory behind this algorithm can be found in the original work by Kantelhardt et al. (2002). A step-by-step implementation of the MFDFA program using Matlab® was given by Ihlen (2012). We made use of the recommendations from the Kantelhardt et al. (2002) and Ihlen (2012) to write a *Python®* program to perform the MFDFA on exhaled breath time signals. Briefly, the algorithm involves dividing the time series data into time intervals of equal length, then applying detrended fluctuation analysis (DFA) (Peng et al. (1994)) to each time interval to remove the trend and then calculating the fluctuation function F. Next, the $q-$order fluctuation function F(q) is obtained by raising the detrended fluctuation function to the power of q. The $q-$order Hurst exponent H(q) is obtained from the scaling behavior of F(q). Then, the algorithm involves estimating the $q-$order mass exponents $\tau(q)$ from $q-$order Hurst exponent H(q), converting them into the $q-$order singularity exponents α, and then computing the generalized singularity dimensions, also known as the singularity spectrum $f(\alpha)$.

In the context of multifractal analysis, a measure of complexity of a time series is its singularity spectrum $f(\alpha)$, which characterizes the distribution of fractal dimensions or scaling exponents α for a signal. While conventional DFA (Peng et al. (1994)) quantifies the average correlation properties of a signal purely through the scaling exponent α, MFDFA provides another important measure. The width of the multifractal spectrum ω (see Fig 4 in main manuscript), which indicates the richness of multifractality present in the experimental data adds further insight into the data. Third-order polynomial fits were used to detrend data in each time interval. The time interval (window) sizes range between 10 and $N/4$

*Corresponding author: mvp@iitm.ac.in

data points, where N is the length of the time series. The orders q of fluctuation function ranges from -5 to 5. It is to be noted that the input time series to the analysis was first normalized, which is discussed in a subsequent section. The chosen normalization method does not alter the compact support of the input time series. The term 'compact support' refers to the property of a time series being nonzero only within a specific time interval. It is essential for a time series to have compact support for reliable multifractal analysis (Kantelhardt et al. (2002)).

Fig 1 consists of a set of plots showing the effect of random shuffling of the exhaled breath time signal on the multifractal singularity spectrum. Figs 1A and 1B show the original and shuffled time series, respectively. The inset plots in each of these plots display the zoomed-in view of the first 1000 data points. It is clearly visible that the existing correlation is destroyed when the data is shuffled. The distribution of the visualised time signal is shown in the form of a histogram in Fig 1C.

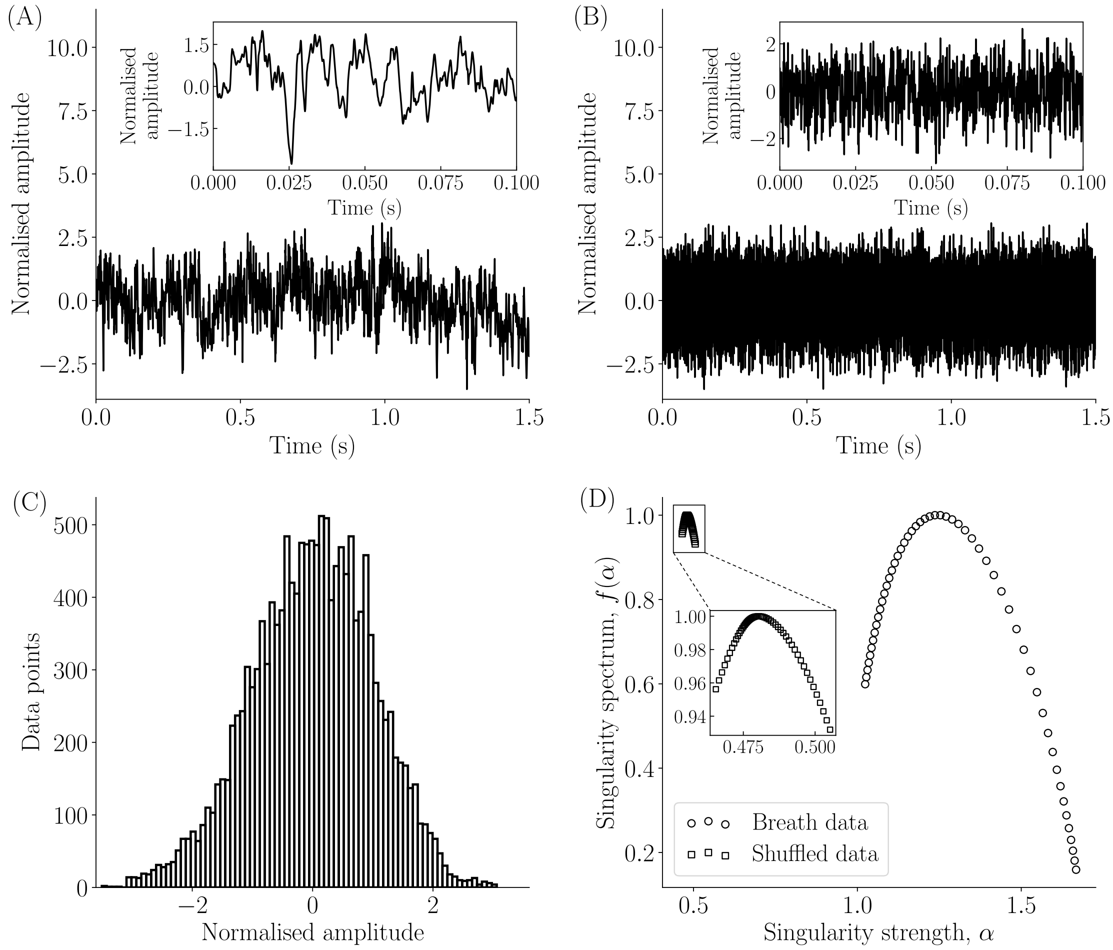

Figure 1: **Comparison of multifractality of time signals.** Plots showing the effect of random shuffling of exhaled breath time series acquired using a hot wire anemometer. Signals shown in (A) and (B) correspond to the actual breath data and the shuffled data respectively. Inset plots in (A) and (B) show a zoomed-in view of the first 1000 data points of the signals. Note that the signal has been normalized using its mean and standard deviation. (C) Histogram showing the distribution of all N data points of the breath signal. (D) Multifractal spectra for the original breath signal and the randomly shuffled white noise signal. Random shuffling causes loss of memory within the time series and losses the multifractality.

The Kolmogorov–Smirnov test for normality (Massey (1951)) revealed that a large fraction of the available breath signals were non-Gaussian. Deviations from a Gaussian or symmetric distribution may be a sign of multifractality stemming from a broad probability distribution function (PDF) as described by Kantelhardt et al. (2002). Shuffling the time series helps us in discovering the reason for multifractality in this case. By randomly permuting the order of values in the time series, temporal correlations are disrupted while preserving the PDF. If the multifractality persists in the shuffled or surrogate data, it suggests that the broad PDF is the primary source of multifractality. Conversely, if the multifractality

disappears in the shuffled data, it indicates multifractality due to inherent long-range temporal correlations. Fig 1D is a plot of the singularity spectral function $f(\alpha)$ against the singularity strength α, resulting from the MFDFA on the time series from 1A and 1B. The plot consists of two representative multifractal spectra - one for the exhaled breath time series and the other corresponding to the same time series shuffled, which becomes a white noise. The white noise signal was observed to form only a tiny arc clustered around $\alpha = 0.5$, while the multifractal breath signal forms a well-defined spectrum. This observation is evidence of the presence of long-range correlations in the breath time signal. Any memory of the correlations (strong or weak) within the time series is lost when randomly shuffled. The inset plot in Fig 1D shows a magnified view of the spectrum from the white noise signal. It can be inferred from this observation that the white noise signal does not show any degree of multifractality and also reconfirms that the multifractality of exhaled velocity is defined by its inherent long-range correlation properties, both for short- and long-range fluctuations. The multifractal analysis was made use in the time series segmentation and feature extraction, which has been discussed in the main manuscript.

Part 2: Model library building procedure and model selection

Following the discussions from the subsection titled *Building of model library* in the main manuscript, generating ${}^{n}C_2$ binary classifiers is necessary to handle the multiclass problem. Let us have a detailed discussion on the model-building procedure and the choice of a binary classifier. We required a detailed analysis since the choice of a classifier depends on the specific characteristics of the dataset and the multiclass problem at hand. The training dataset was used to construct binary classifier models for each user-pair. Decision tree (DT), random forest (RF), support vector machine (SVM), logistic regression (LR), Gaussian naive Bayes (GNB), and multi-layer perceptron (MLP) were chosen as the candidate binary classifier models. The machine learning models employed in our study are discriminative models except for one, the GNB. Discriminative models do not try to identify the distribution that generates the data; instead, they try to find out the features that separate classes from each other (Ng and Jordan (2001)).

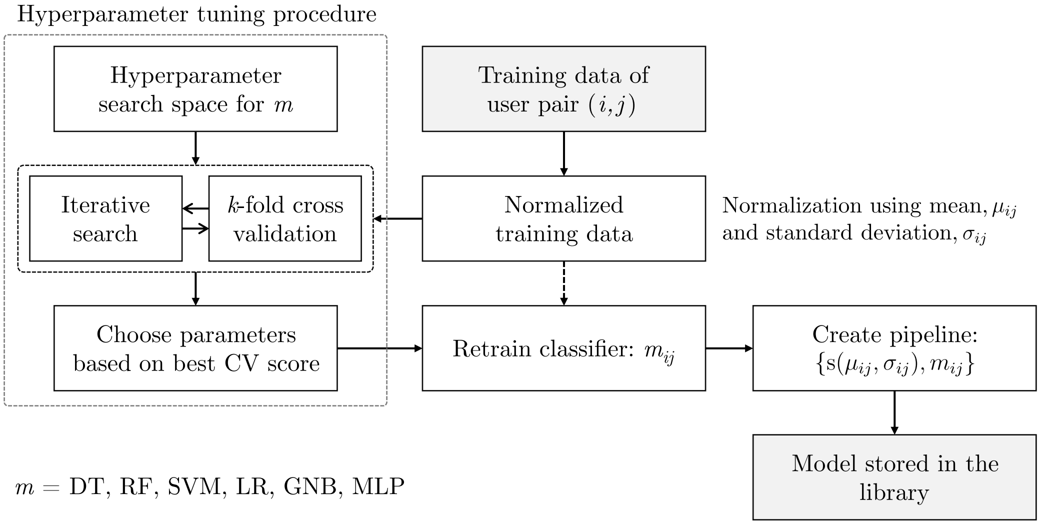

Figure 2: **Model library building procedure.** Flow chart showing the model library building procedure for a user-pair (i, j), where $i = 1, 2, \ldots n$; $j = 1, 2, \ldots n$; n is the total number of users. Note that model $m_{ij} \equiv m_{ji}$, and therefore, only model m_{ij} are built and stored in the library. The abbreviations stand for the following: CV - cross validation, DT - decision tree, RF - random forest, SVM - support vector machine, LR - logistic regression, GNB - Gaussian naive Bayes, MLP - multi-layer perceptron. $s(\mu_{ij}, \sigma_{ij})$ is the standard scaling function, μ_{ij} and σ_{ij} are the mean and standard deviation respectively of the training data of users i and j combined.

Robustness of model parameters and selection of the best model is very crucial to the performance of a user authentication algorithm. Optimal tuning improves the generalizability of each machine learning model. A generic algorithm for hyperparameter tuning and model selection is illustrated in Fig 2. The training data for a user-pair (i, j) is normalised initially using the training set's mean (μ_{ij}) and standard

deviation (σ_{ij}). μ_{ij} and σ_{ij} should be stored in the memory as it is required for scaling the test data when required. Hence, it can be combined into a function called standard scaling function $s(\mu_{ij}, \sigma_{ij})$ for later use. This normalised training data is now used for tuning and training the best model. It is generally challenging to know the values of the model parameters for a given machine learning model on a dataset. Therefore, we have employed an iterative search cross-validation scheme to compare different sets of hyperparameter values for each model. A stratified k-fold cross-validation technique with hyperparameter tuning was employed for the evaluation and selection of the model parameters. The number (k) of folds was chosen to be 5. Parameters from a hyperparameter search space were fed into the cross-validation algorithm, where the training data was split into k equally sized folds, maintaining the same target class distribution in each fold as the original dataset. This will make sure that there is no class imbalance in each of the k folds. The iterative search from the search space was performed either using Bayesian optimisation based search (Bergstra et al. (2011), Bergstra et al. (2013)) or by grid search depending on the size of the search space of a classifier model. The Bayesian search method employs a probabilistic model of the search space to choose a hyperparameter configuration. By combining exploration (experimenting with new configurations) and exploitation (utilizing knowledge from previous iterations), it effectively explores the hyperparameter space and identifies promising regions in the hyperparameter space, as described by Snoek et al. (2012). The grid search can be described as an exhaustive exploration method which tests all the combinations of the search space (Belete and Huchaiah (2022)). Bayesian search was employed when the search space was large, whereas the grid search was employed for smaller search space where the method of brute force was computationally affordable. During an iteration, for a selected hyperparameter configuration, the model was trained and evaluated k times, each time using a different fold as the validation set and the remaining $k-1$ folds as the training set. The set of parameters which yielded the best cross-validation score were selected, and the model was retrained on the normalised training data based on the selected parameters. The standard scaling function and the classifier model are combined into a model pipeline $\{s(\mu_{ij}, \sigma_{ij}), m_{ij}\}$, where m_{ij} is the classifier built corresponding to user-pair (i, j). The pipeline was created to make sure that the test data, when introduced, should be scaled using the training set's mean and standard deviation before predictions are made.

The above procedure was performed for all the candidate binary classifiers. This procedure helped in effectively reducing the size of the feature set from ≈ 450 dimensions to 10 dimensions using random forest classifiers as briefed in the subsection titled *Feature extraction* in the main manuscript. The number of trees/estimators was tuned. More trees are generally required for the purpose of refined variable importance estimations, as noted by Genuer et al. (2010). The rule of splitting was tuned by controlling the maximum depth of a tree, minimum number of samples required to split an internal node, and minimum number of samples required to be at a leaf node. It was important to figure out the best model based on their performance. This will allow us to build an efficient library of best estimators for the given training data. Each of the ${}^nC_2$ models underwent a hyperparameter tuning before being fit to the training data. This way it was ensured that each model is generalized for the corresponding user-pair's data. It is important to note here that any model with a cross-validation score of 60% or less was discarded. This was done to ensure that the models which were saved in the library are far from a random model. Hence, by storing only the models with a cross-validation score above 60%, we make sure that the overall algorithm performs between reasonably well to good, based on the generalisation of the models built.

Selection of the best binary classifier model for a given user-pair can be made by picking the model with the highest cross-validation score. This technique can be called as *best-of-all* model selection technique. Fig 3A shows the percent proportion of different models in a library which was built using this procedure. We observed that MLPs were the most frequently occurring best classifier based on the highest cross-validation score. The second frequently occurring one being the RF, followed by SVM, LR, DT and GNB. The process of building all 6 models and choosing the best one every time is computationally very expensive, So, one out of the 6 classifiers had to be chosen for testing the algorithms. The information from Fig 3A is not sufficient to make this decision, since a good cross-validation score does not always promise a good performance on test data since the classifiers could also be overfitting the training dataset in certain cases. Pairwise user test data was used to test each candidate classifier and the results are visualised as a box and whiskers plot in Fig 3B. The orange line inside the boxes represents the median of the test score. It is clear that DT and GNB classifiers perform poorer than the others, and, RF, SVM, LR and MLP have very similar performance on the test data. All the models have produced test accuracies ranging from very low values below 0.5 to 1. Looking at the outliers data points, we can see that SVM does very poorly as the accuracy even goes below 0.2, and in fact LR and MLP too have produced accuracies below 0.2. The RF classifiers and GNB have similar lower bound of test accuracy.

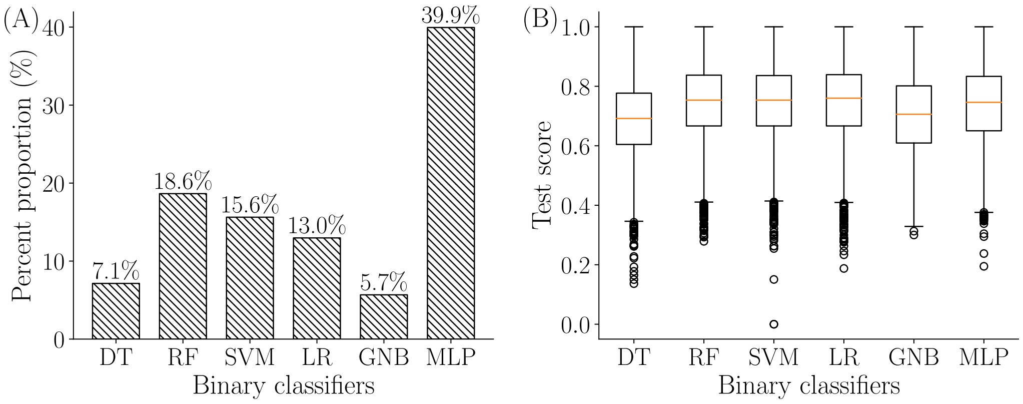

Figure 3: **Comparison of candidate classifier models.** (A) Bar chart showing the percent proportion of each model in the library in the case of *best-of-all* model selection procedure. (B) Box and whiskers plot showing the spread of test accuracy of each classifier. The orange line inside the boxes represent the median.

In order to get a better understanding on how these models fit the training data, we can visualise the decision boundaries in a 2D feature space. Since we are already working on a reduced feature space of 10 dimensions, choosing any 2 dimensions out of it and building models for the purpose of visualisation seems appropriate here. The (β, ω) space was chosen for visualisation. To generate the 2D decision boundaries, a structured synthetic dataset was generated which filled up the two-dimensional feature space within the given bounds. The decision regions are obtained based on the predictions made on each data point from the synthetic dataset. These boundaries are visualised for comparison in Figs 4A−4R for three randomly chosen user-pairs.

A region R in the feature space is classified as a decision region under class y_i $(i = \{0, 1\})$ if all the samples x_j in that region are classified as y_i. A decision boundary separates these 2 decision regions. Therefore, it can be observed that the feature space is divided into two parts by the decision boundary for a binary classification problem. Such a representation not only helps us visualize the difference between two classes, but also helps us in comparing multiple binary classifiers and their decision mechanisms. The scattered points in each plot of Fig 4 represent the training data points with their respective colors corresponding to two users. The test data accuracy for each model is displayed at the top right corner of their respective plots. Considering user-pair X (Figs 4A−4F), all the models were able to perform well, with LR and RF producing the scores higher than the other choices. Similarly, for user-pair Y (Figs 4G−4L), all models were able to perform well, with GNB, MLP and RF producing the best scores. It is interesting to see that the decision boundaries captured by SVM (Fig 4H) and RF (Fig 4K) appear similar but small variations in the captured boundary causes one algorithm to perform better. Considering user-pair Z (Figs 4M−4R), the models produce less accuracy against the test data when compared with user-pairs X and Y. GNB and RF produce the highest scores among the models. From the discussion so far, it is evidenced that the random forest models (see Figs 4B, 4H and 4N) were able to capture a complex decision boundary in all the three cases and are able to perform well in all the three cases. This could be due to its bootstrapping and ensemble schemes, making it robust to outliers. Also, RF is known to reduce the risk of overfitting by aggregating predictions from multiple decision trees and generalising well. Therefore, we chose random forest as the apt binary classifier model for the model library. All the ${}^{n}C_2$ trained models (the pipelines, as discussed earlier) were stored in the library, where n is the total number of users.

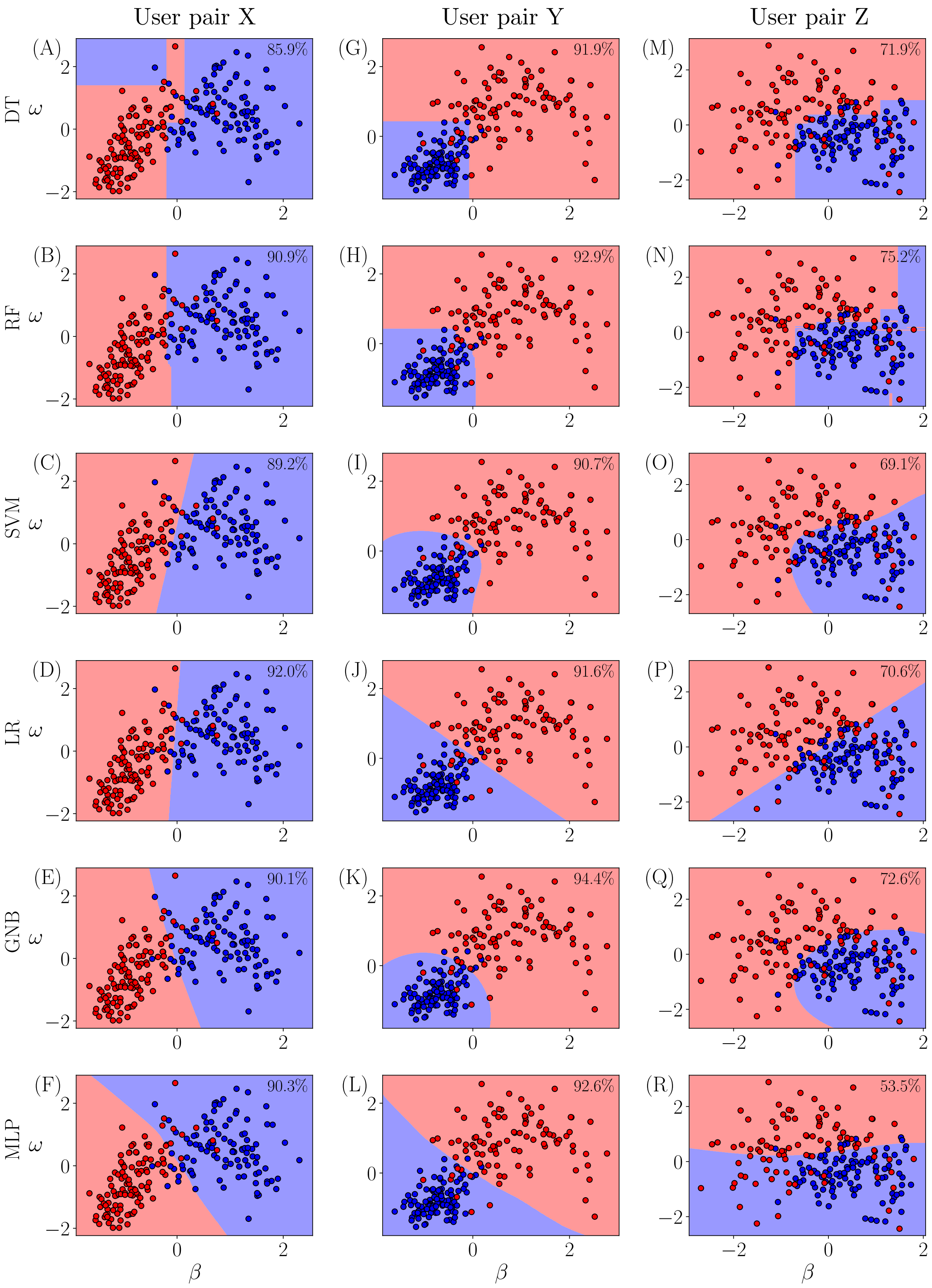

Figure 4: **Two dimensional decision boundaries.** Comparison of two dimensional decision boundaries in the (β, ω) plane, captured by different models for three randomly chosen user-pairs. The scattered points are the training data points with red and blue labels denoting their true classes respectively. The line separating the two contour regions is the decision boundary. Accuracy of each model against the test data is displayed at the top right corner of their respective plots. The abbreviations stand for the following: DT - decision tree, RF - random forest, SVM - support vector machine, LR - logistic regression, GNB - Gaussian naive Bayes, MLP - multi-layer perceptron.

References

Belete, D.M., Huchaiah, M.D., 2022. Grid search in hyperparameter optimization of machine learning models for prediction of hiv/aids test results. International Journal of Computers and Applications 44, 875–886.

Bergstra, J., Bardenet, R., Bengio, Y., Kégl, B., 2011. Algorithms for hyper-parameter optimization, in: Shawe-Taylor, J., Zemel, R., Bartlett, P., Pereira, F., Weinberger, K. (Eds.), Advances in Neural Information Processing Systems, Curran Associates, Inc.

Bergstra, J., Yamins, D., Cox, D.D., 2013. Making a science of model search: Hyperparameter optimization in hundreds of dimensions for vision architectures, in: Proceedings of the 30th International Conference on International Conference on Machine Learning - Volume 28, JMLR.org. p. I–115–I–123.

Genuer, R., Poggi, J.M., Tuleau-Malot, C., 2010. Variable selection using random forests. Pattern Recognition Letters 31, 2225–2236.

Hurst, H.E., 1951. Long-term storage capacity of reservoirs. Transactions of the American Society of Civil Engineers 116, 770–799.

Ihlen, E., 2012. Introduction to multifractal detrended fluctuation analysis in matlab. Frontiers in Physiology 3.

Kantelhardt, J.W., Zschiegner, S.A., Koscielny-Bunde, E., Havlin, S., Bunde, A., Stanley, H.E., 2002. Multifractal detrended fluctuation analysis of nonstationary time series. Physica A: Statistical Mechanics and its Applications 316, 87–114.

Massey, F.J., 1951. The kolmogorov-smirnov test for goodness of fit. Journal of the American Statistical Association 46, 68–78.

Ng, A.Y., Jordan, M.I., 2001. On discriminative vs. generative classifiers: A comparison of logistic regression and naive bayes, in: Proceedings of the 14th International Conference on Neural Information Processing Systems: Natural and Synthetic, MIT Press. p. 841–848.

Peng, C.K., Buldyrev, S.V., Havlin, S., Simons, M., Stanley, H.E., Goldberger, A.L., 1994. Mosaic organization of dna nucleotides. Phys. Rev. E 49, 1685–1689.

Snoek, J., Larochelle, H., Adams, R.P., 2012. Practical bayesian optimization of machine learning algorithms, in: Proceedings of the 25th International Conference on Neural Information Processing Systems - Volume 2, Curran Associates Inc.. p. 2951–2959.

Sreenivasan, K.R., 1991. Fractals and multifractals in fluid turbulence. Annual Review of Fluid Mechanics 23, 539–604.
